# Active surveillance for low-grade appendiceal mucinous neoplasm (LAMN)

**DOI:** 10.1515/pp-2023-0032

**Published:** 2023-12-29

**Authors:** Christian Mouawad, Armelle Bardier, Mathilde Wagner, Solène Doat, Dahbia Djelil, Jade Fawaz, Marc Pocard

**Affiliations:** Department of Digestive, Hepatobiliary and Liver Transplantation Surgery, AP-HP, Hôpital de la Pitié-Salpêtrière, Paris, France; Department of Pathology, AP-HP, Hôpital de la Pitié-Salpêtrière, Paris, France; Department of Radiology, AP-HP, Hôpital de la Pitié-Salpêtrière, Paris, France; Department of Gastroenterology, AP-HP, Hôpital de la Pitié-Salpêtrière, Paris, France; Université Paris Cité, INSERM, U1275 CAP Paris-Tech, Paris, France

**Keywords:** appendix, low-grade mucinous neoplasm, active surveillance

## Abstract

**Objectives:**

Due to the scarcity of low-grade appendiceal mucinous neoplasm (LAMN), there is an absence of systematized guidelines concerning its management, especially after incidental finding on an appendiceal specimen. In this study, we evaluate the active surveillance (AS) strategy adopted for a series of patients diagnosed with LAMN on resection specimens who were considered to have a low risk of pseudomyxoma progression.

**Methods:**

Thirty patients were included between April 2014 and July 2021, with a female majority and a median follow-up period of 3.1 years. The inclusion criteria were as follows: LAMN diagnosis on appendiceal specimens, confirmed in an expert center, limited extra-appendiceal mucin resected and localized around the appendix, normal biology (CEA, CA199, CA125) and normal abdominopelvic MRI. AS included physical exam (trocar scar), biology and MRI, 6 months postoperatively, then yearly for 10 years.

**Results:**

As an initial surgery, 77 % had an appendectomy as their initial intervention, 17 % had a cecectomy, and 6 % had a right colectomy. After follow-up, 87 % of patients showed no sign of disease progression by MRI, while 13 % progressed to PMP. MRI performed in the first postoperative year predicted the disease prognosis in 97 % of patients.

**Conclusions:**

The AS strategy, based on MRI, is a valid option after incidental LAMN diagnosis.

## Introduction

Appendiceal tumors are infrequent, representing approximatively 0.5 % of all tumors of the gastrointestinal tract [1]. While neoplasms may be found in 0.9–1.7 % of appendectomy specimens [2, 3], almost half of them can be characterized as low-grade appendiceal mucinous neoplasms (LAMNs) [4]. The term LAMN was suggested in 2003 by Misdraji et al. [5] and then accepted by the World Health Organization (WHO) [6]. These neoplasms are marked by an appendix filled with mucin secondary to partial epithelial replacement with low-grade neoplastic epithelial glandular cells presenting diverse stages of mucosal atrophy and mural fibrosis [7]. Mucinous or epithelial penetration in the serosa exposes the patient to the risk of pseudomyxoma peritonei (PMP) development [8], with no hematogenic or lymphogenic metastases [9], [10], [11], [12]. Due to the scarcity of LAMN, there is an absence of systematized guidelines concerning its management, especially after incidental finding on an appendiceal specimen. The discussed therapeutic procedures vary among conservative surveillance, cecectomy, ileotyphlectomy, right colectomy, and in advanced cases, cytoreductive surgery (CRS), peritonectomy, and hyperthermic intraperitoneal chemotherapy (HIPEC). This report describes our experience with the active surveillance (AS) strategy adopted for a series of patients diagnosed with LAMN.

## Materials and methods

The decision to start an AS program was decided in 2014. The decision was related to the ongoing discussion regarding the secondary effects and postoperative complications in cases of CRS and HIPEC, balanced with a limited risk of disease progression to PMP. We prospectively recorded data from patients diagnosed with LAMN on resection specimens who were referred to our expertise unit for secondary opinion and were included in the active surveillance strategy. All data were anonymously collected, and according to the “Loi Jardé” (French law amended by Order no. 2016-800 and its implementing decree no. 2016-1537 of 16/11/2016 related to research involving the human person), no patient consent was needed, as the treatment implemented in this study was considered one of the standard recommended therapies.

All cases were validated to receive an active surveillance procedure by the local tumor board if they met the inclusion criteria and if no CRS or HIPEC were proposed. The inclusion criteria were as follows: LAMN diagnosis on appendiceal specimens, confirmed in an expert center, limited extra appendix mucin resected (acellular and cellular) and localized around the appendix (including the appendix wall, cecum, adjacent peritoneum) or at distant sites (including the bladder, ovaries, distal sigmoid, pouch of Douglas), normal biology (CEA, CA199, CA125) and normal abdominopelvic MRI. AS included physical examination (trocar scar), biology and MRI 6 months after appendectomy and then yearly for 10 years.

Prospective records were used to identify LAMNs diagnosed on appendiceal specimens managed between April 2014 and July 2021. We reviewed data including age, sex, diagnosis date, tumor marker levels [carcinoembryonic antigen (CEA), cancer antigen 125 (CA-125), CA19-9], date and type of surgical intervention, pre- and postoperative imaging reports, operative reports, pathology reports, and multidisciplinary conference reports.

A review of all clinical, biological and imaging records was performed to identify cases of local recurrence or peritoneal dissemination (i.e., PMP). The follow-up duration was defined as the time from the initial surgery before the LAMN diagnosis to the time of the last available MRI report, last abdominal intervention, or the last available clinical assessment by the surgeon. All included patients had at least 1 year of follow-up.

Operative reports were reviewed for the extent of resection, perforation signs, mucin dissemination, and suspicion of appendiceal base involvement. Pathology reports were searched for proximal margin positivity, presence of perforation, and presence of cellular or acellular extraluminal mucin. Disease recurrence was defined by the presence of suspicious implants on MRI reports during follow-up or at the time of a subsequent surgery. Inclusion criteria were defined as follows: LAMN diagnosis on appendiceal specimens, confirmed in expert center, limited extra appendix mucin resected and localized around appendix, normal biology (CEA, CA199, CA125) and normal abdominopelvic MRI. Exclusion criteria were included the following: a different appendiceal neoplasm, initial PMP diagnosis, incomplete data at the time of diagnosis, and loss to follow-up in less than a year.

Descriptive statistics are presented as percentages for categorical variables and as the means and standard deviations (SD) for continuous variables, performed using IBM^®^ SPSS^®^ software SPSS Inc., IBM (International Business Machines Corporation), Chicago, Illinois, United States Statistics for Windows version 21.

## Results

Of the 55 patients listed initially with LAMN diagnosis between April 2014 and July 2021, 30 patients were included in our study after the exclusion criteria were applied. Clinical data are shown in Table 1.

**Table 1:** Patient characteristics.

Characteristics	Number of patients, %
All	30 (100)
Sex: Female	19 (64)
Sex: Male	11 (36)
Initial surgery: Appendectomy	23 (77)
Initial surgery: Cecectomy	5 (17)
Initial surgery: Right colectomy	2 (6)
Surgical approach: Laparoscopy	27 (90)
Surgical approach: Open technique	3 (10)
Perioperative findings: Appendiceal perforation	12 (40)
Perioperative findings: Regional mucin	13 (43)
Perioperative findings: Distant mucin	3 (10)
Perioperative findings: Involvement of appendiceal base	1 (3.3)
Histological findings: Appendiceal perforation	16 (53)
Histological findings: Proximal-margin involvement	2 (6)
Histological findings: Acellular mucinous deposits	4 (13)
Histological findings: Cellular mucinous deposits	1 (3.3)
Disease progression	4 (13)

In our case series, 19 female patients (64 %) and 11 male patients (36 %) were included. The median age of the patients at the time of diagnosis was 52±18.5 years, ranging from 18 to 81 years. The median follow-up duration was 3.1±1.5 years. Twenty-three patients (77 %) had an appendectomy as their initial intervention, five patients (17 %) had a cecectomy, and two patients (6 %) had a right colectomy. Twenty-seven patients (90 %) underwent an initial laparoscopic procedure, while three patients (10 %) underwent an open technique (right colectomy for two patients and cecectomy for one patient).

During the initial surgical procedure, appendiceal perforation was noted by the surgeon in 12 patients (40 %), while the presence of regional mucin (including the appendix wall, cecum, and adjacent peritoneum) was described in 13 patients (43 %), and the presence of mucin at distant sites (including the bladder, ovaries, distal sigmoid, and pouch of Douglas) was described in three patients (10 %). Involvement of the appendiceal base was noted in only one case (3.3 %); therefore, a cecectomy was performed.

Histologically, appendiceal perforation was described in 16 patients (53 %). Proximal margin involvement was found in two patients (6 %). Acellular mucinous deposits were found in four patients (13 %), and cellular mucinous deposits were found in one patient (3.3 %).

**Figure 1:**
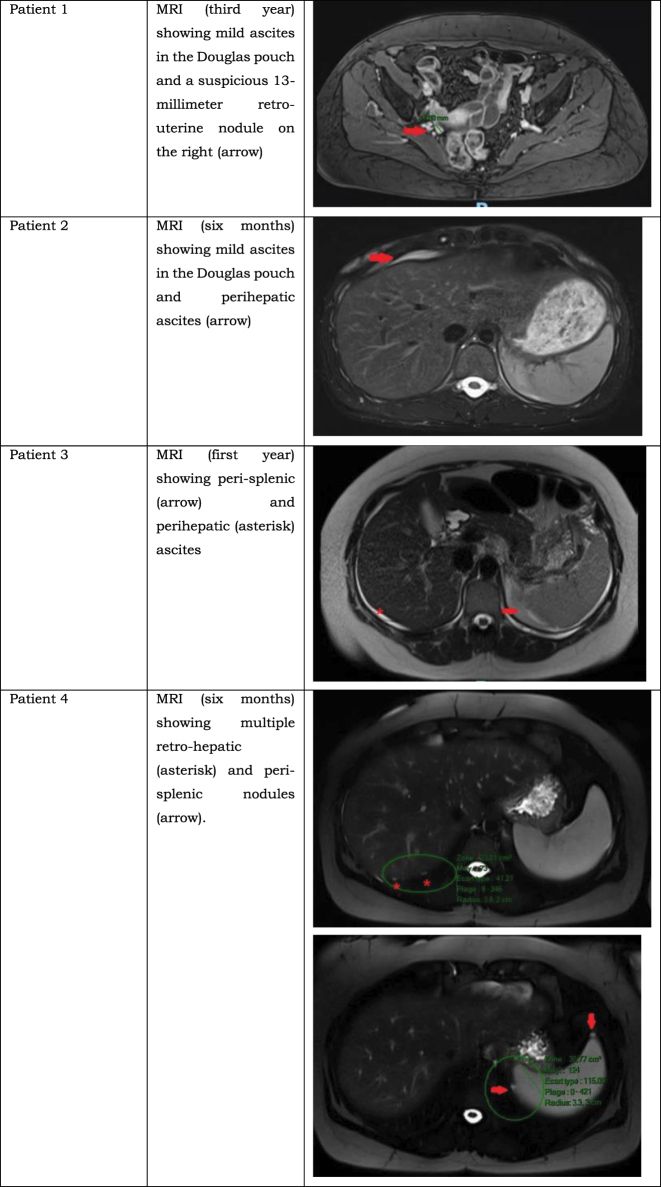
Suspicious lesions seen on surveillance MRI suggesting disease progression.

The decision of an AS strategy was directly proposed by the surgeon in 10 patients (33 %) and then confirmed by the tumor board; the decision was secondary to the tumor board discussion in 20 patients (67 %). Concerning the tumor markers (CEA, CA19-9, CA-125), we identified no elevation in the serum levels postoperatively or during the entire follow-up period in any of the patients. Colonoscopic monitoring was performed in five patients (17 %) with negative results. For the follow-up imaging results, patients underwent a yearly MRI for surveillance. MRI was performed in an expert center in 25 patients (83 %). After a median follow-up period of 3.1 years, 26 patients (87 %) showed no sign of PMP on imaging results, while four patients (13 %) showed lesions compatible with disease progression on surveillance MRI (Figure 1). Out of these four patients, three patients underwent cytoreductive surgery associated with HIPEC after a collective decision by a tumor board consultation, and the fourth patient was waiting for her scheduled intervention. The outcomes in these patients are shown in Table 2.

**Table 2:** Outcome of patients who showed disease progression during active surveillance.

	Time of diagnosis of lesions on MRI suspicious of progression	CRS + HIPEC	Peritoneal cancer index score (PCI) found during CRS + HIPEC	No residual peritoneal disease after CRS (CC 0)	Outcome after CRS + HIPEC	Surveillance post CRS + HIPEC
Patient 1	Third year	Yes	2/39	Yes	Asymptomatic incisional hernia repaired one year post surgery	MRI done at one year post CRS showing no sign of disease progression
Patient 2	Six months	Yes	5/39	Yes	Hemoperitoneum at postoperative day nine: laparotomy, ileocecal resection	MRI done at six years post CRS suggesting a disease progression
Patient 3	First year	Yes	15/39	Yes	Disease progression shown on MRI done at 30 months postsurgery: a redo CRS+HIPEC was done 3 years after the first one (PCI: 18/39)	MRI done at four years post redo-surgery showing no sign of disease progression
Patient 4	Six months	No	Not applicable	Not applicable	Not applicable	CRS+HIPEC scheduled in the future

All four patients who showed disease progression underwent an initial laparoscopic approach, with an appendectomy performed for three of them and a cecectomy for the fourth patient due to suspicion of appendiceal base involvement during the intervention. Half of them showed a suspicion of perforation perioperatively. Additionally, half of them showed a suspicion of regional mucinous implants. In the pathology reports, appendiceal perforation was described in three out of four patients, with the presence of acellular mucinous implants in one patient and cellular mucinous implants in another patient. For the surveillance imaging, three out of the four patients who progressed to PMP had an MRI performed in the first postoperative year that showed a suspicion of disease progression (localized ascites, peritoneal implants or nodules, etc.). The fourth patient did not show any suspicious features on his yearly MRI until his third postoperative year.

## Discussion

While LAMN can be considered a morphologically benign tumor, it confers a risk of aggressive progression to PMP, which raises the question of the management of LAMN diagnosed on a surgical specimen resected following various presentations: acute appendicitis, incidental finding of mucocele on imaging, and suspicion of ovarian mass. Disease progression is rare with intact removal of mucocele, and many arguable risk factors may expose the patient to PMP, such as LAMN, appendiceal perforation, positive resection margins, and extraluminal mucinous deposits [8, 9, 13], [14], [15]. After LAMN diagnosis, an appendectomy would seem adequate for a tumor limited to the appendix with no extension [16], while a right hemicolectomy or cecectomy was suggested when peri-appendicular tumoral extension was found [8, 17]. However, no amelioration in prognosis could be found when comparing patients who underwent right hemicolectomy with those who underwent appendectomy [18]. In patients diagnosed with PMP, a 40 % mortality rate was estimated within 10 years [19], for which HIPEC and CRS are considered the standard management strategies [19], [20], [21].

In our case series, the majority of patients were females (64 %), consistent with many studies showing a predominance of women among patients diagnosed with LAMN [13, 22]. The vast majority (90 %) underwent their initial surgery by the laparoscopic approach, while only 10 % underwent an open technique procedure due to the presence of a peri-appendicular abscess or adjacent mucin, which needed conversion for complete resection. For the type of intervention, the majority underwent appendectomy (77 %), while 17 % had a cecectomy due to suspicion of periappendicular mucin deposits or to suspicion of appendiceal base involvement. Right colectomy was performed in 6 % of cases where perforation was found with intestinal adherence to the appendix or suspicion of mucin deposits alongside the right paracolic gutter.

On histological examination, appendiceal perforation was described in 53 % of patients, almost identical to the percentage (52 %) reported by Fournier et al. [7]. Proximal margin involvement was found in 6 % of patients. Acellular mucinous deposits were found in 13 % of patients and cellular mucinous deposits were found in 3.3 % of patients. All of the latter were previously described as risk factors for disease progression [8, 9, 13], [14], [15], which would typically lead to a surgical intervention for a more extended resection with the possibility of HIPEC [12]. However, this strategy is highly controversial and is presented in total contrast to the conclusion of some scholars that surveillance post LAMN resection is unnecessary [23]. Consequently, there is still no standardized care in cases of LAMN diagnosed on surgical specimens, taking into consideration the high morbidity rate of surgical reintervention with no proven benefits in the face of possible disease progression. Therefore, based on our experience, we evaluated the AS strategy for a series of 30 patients.

In our surveillance strategy, taking into consideration that two out of the four patients who progressed to PMP had missing data concerning their tumor marker levels, we found that tumor marker serum levels showed no abnormalities in any of the patients who had postoperative dosages. However, many studies have shown that LAMN prognosis is related to serum tumor marker levels [7, 13], while scholars in other studies doubted its contribution to diagnostic specificity outside possible usage in the follow-up of tumors with proven peritoneal involvement [24]. Therefore, tumor marker levels may be considered an unreliable tool for LAMN surveillance based on the debatable data we found in the current literature.

For imaging surveillance, we chose MRI, which is considered a consensus approach for evaluation in the event of LAMN follow-up according to Delhorme et al. [24]. MRI was performed in an expert center for 83 % of the patients. Our AS strategy emphasizes the systematic use of MRI as a sole radiological exam for surveillance, a matter that is still not highlighted in many published international guidelines tackling the management of LAMN [25, 26]. Our diagnosis of disease progression was mostly based on the MRI results, in which 87 % of the patients showed no sign of disease progression during the follow-up period, while 13 % of patients progressed to PMP. Since three out of the four patients who progressed to PMP showed suspicious features on MRI performed in the first postoperative year, we can deduce that the first postoperative year MRI predicted the prognosis in 29 out of 30 patients (97 %) after a median follow-up duration of 3.1 years. This conclusion is in accordance with a study by Reiter et al. [27], which showed that 20 % of their patients who were followed up after LAMN diagnosis on surgical specimens progressed to PMP in an average of 12.4 months. In our case series, only one patient progressed to PMP, while his yearly MRI (performed in an expert center) showed the absence of suspicious lesions until his third postoperative year. Three out of the four patients diagnosed with PMP underwent cytoreductive surgery associated with HIPEC after a collective decision by a tumor board in accordance with the French Intergroup Clinical Practice Guidelines concerning PMP management [24], and the fourth patient is waiting for her scheduled intervention.

Nineteen percent of patients diagnosed with appendiceal perforation on pathology reports progressed from LAMN to PMP. The notion of perforation as a risk factor for disease progression is debated [13, 14]. Our AS strategy in the face of appendiceal perforation in patients diagnosed with LAMN is in conformity with a study by Guaglio et al. [9], which indicated that appendix wall perforation had no significant association with metachronous peritoneal recurrence. The latter study also showed that mucinous implants carry a low recurrence risk, justifying conservative management when radical resection of LAMN was achieved in initial surgery. In our case series, the single patient who had cellular mucinous neoplasms as well as one of the four patients who had acellular mucinous neoplasms exhibited progression to PMP, which may support the study by Roxburgh et al. [28], who considered the cellularity of mucinous deposits as a determinant prognostic factor in LAMN; however, this does not necessarily justify a surgical reintervention in every patient presenting cellular mucinous deposits. In our series, two patients (6 %) had a positive proximal margin after appendectomy, and after almost two years of follow-up for both patients, no progression of disease was marked on the yearly imaging results. Our AS approach for these patients is in accordance with a study by Ibrahim et al. [29], which supported conservative management in patients diagnosed with LAMN and presenting involvement of the appendectomy margin, but indicates some disregard for the Grade C recommendation of performing a cecectomy following incomplete LAMN resection [24].

### Limitations

Since our institution is a tertiary center for peritoneal disease, many of the pathology slides are transferred from other hospitals, possibly limiting the evaluation of the totality of the appendix and the degree of margin invasion. Additionally, we had some incomplete data concerning some evaluation criteria (e.g., tumor marker levels and colonoscopic findings). Last, another limitation is our limited sample size, which is secondary to the rarity of the tumor.

## Conclusions

Our prospective cohort study including 30 patients with incidental LAMN diagnosis following initial resection showed that the AS strategy is a valid option independent of preoperative findings and histological characteristics. MRI performed in the first postoperative year predicted the disease prognosis in 97 % of patients after a median follow-up duration of 3.1 years. However, further investigation is needed to establish a standardized management plan in cases of LAMN diagnosis, especially given the many debatable risk factors described in pathology reports.
